# Immunomodulatory effect of stress hormones on porcine neutrophil functions during *Actinobacillus pleuropneumoniae* infection

**DOI:** 10.1007/s00430-026-00881-3

**Published:** 2026-07-27

**Authors:** Marta C. Bonilla, Simon Lassnig, Michael Wendt, Isabel Hennig-Pauka, Matthias Mörgelin, Maren von Köckritz-Blickwede, Nicole de Buhr

**Affiliations:** 1 Institute of Biochemistry, University of Veterinary Medicine Hannover, Foundation, Hannover, Germany; 2 Research Center for Emerging Infections and Zoonoses (RIZ), University of Veterinary Medicine Hannover, Foundation, Hannover, Germany; 3 Clinic for Swine, Small Ruminants and Forensic Medicine and Ambulatory Service, University of Veterinary Medicine Hannover, Hannover, Germany; 4 Field Station for Epidemiology, University of Veterinary Medicine Hannover, Bakum, Germany; 5 Colzyx AB, Lund, Sweden

**Keywords:** Neutrophils, Neutrophil extracellular traps (NETs), *Actinobacillus pleuropneumoniae* (*A.pp*), Stress hormones, Host–pathogen interaction, Cortisol, Epinephrine, Norepinephrine, Reactive oxygen species (ROS)

## Abstract

Stress contributes to disease outcomes and can influence the signaling and interactions of immune cells. This study aimed to evaluate the influence of three selected stress hormones cortisol, epinephrine and norepinephrine, at in vivo-relevant concentrations, on the antimicrobial activity of porcine neutrophils during infection with *Actinobacillus pleuropneumoniae* (*A.pp*). We analyzed whether stress hormones affect the antimicrobial activity of neutrophils. Reactive oxygen species (ROS) production, cell size, and granularity were measured using flow cytometry. Neutrophil extracellular trap (NET) formation was quantified using confocal immunofluorescence microscopy, and transmission electron microscopy was employed to analyze the formation of vesicular NETs. ROS production and NET formation experiments were also conducted under infection with *A.pp*. The survival of *A.pp* in the presence of stress hormone-treated neutrophils was assessed. The antimicrobial activity of neutrophils was altered in a concentration-dependent manner by all tested stress hormones: neutrophils produced higher amounts of ROS and released significantly more NETs under *A.pp* infection, in particular vesicular NET formation. However, *A.pp* growth in the presence or absence of neutrophils was not affected by stress hormones. Future studies are needed to characterize the role of vesicular NETs as stress response reactions. In conclusion, this study contributes to a better understanding of the innate immune cells, particularly porcine neutrophils, in infections within stressed hosts.

**Supplementary Information:**

The online version contains supplementary material available at 10.1007/s00430-026-00881-3.

## Introduction

The term "stress" refers to the disruption of homeostasis, which hinders the maintenance of a normal physiological state [1]. It occurs when a stimulus triggers a “fight-or-flight reaction” in the brain, leading to physical stress responses in the body [2]. Stress can be classified into two types based on the duration of stimulation: acute stress and chronic stress. Acute stress is generally considered beneficial for survival, while chronic stress is often recognized as harmful to health [3]. Prolonged exposure to stress affects the health and behavior of both humans and animals, particularly evident during chronic stress. This can lead to allostatic overload, which increases the risk of physiological disorders and the occurrence of pathological changes [4]. Stress is not only a consequence but also a potential trigger of infectious diseases [5]. It can alter the secretory response of neuroendocrine mediators, impacting the signaling and interactions of immune cells [6]. Despite efforts to improve the husbandry conditions of livestock animals [7], they are still subjected to various stressful stimuli, which can influence the progression and outcome of diseases [8, 9].

Among the key mediators of the stress response are the hormones cortisol, adrenaline (epinephrine), and noradrenaline (norepinephrine). These hormones may critically influence the innate immune system by modulating the activity of key effector cells such as neutrophils. Under stress conditions, their dysregulated release can impair pathogen clearance, alter cytokine profiles, and compromise barrier defenses[2, 10, 11]. This highlights the pivotal role of neuroendocrine-immune interactions in shaping early immune responses and influencing disease susceptibility. Detrimental stress, being a systemic condition, affects the organism immune system in multiple ways and locations, thereby decreasing its resistance against infectious diseases [5, 12]. This study, however, focuses on the effects of acute stress on the innate immune system, particularly on neutrophil granulocytes.

During bacterial infections, neutrophils serve as the first line of defense against pathogens [13]. Their antimicrobial mechanisms include phagocytosis, the production of reactive oxygen species (ROS), and the release of neutrophil extracellular traps (NETs) [14]. NETs are extracellular chromatin structures composed of a DNA backbone adorned with antimicrobial components [15].They can be released through two processes: "suicidal" NETosis and "vital" NETosis. In "suicidal" NETosis, the nucleus decondenses, allowing granular proteins to interact with and adhere to the DNA [16, 17]. This process causes the cell membrane to rupture, releasing NETs into the extracellular space, ultimately resulting in the death of the neutrophil [18, 19]. In contrast, during "vital" NETosis, vesicles containing DNA from the nucleus and antimicrobial components from the granules migrate to the cell membrane [20–22]. These vesicles are released into the extracellular space while the neutrophil's cell membrane remains intact, allowing the neutrophil to continue with other defense mechanisms [23]. NETs immobilize, eliminate and reduce the spreading of pathogens [24]. Increasing evidence suggests that NETs are not only involved in clearing pathogens but also have both direct and indirect regulatory effects on innate and adaptive immune responses. This highlights their key in maintaining immune homeostasis [25].

Neutrophils are highly sensitive to stress-induced changes, which can affect their distribution in the body, functional capacity, and lifespan, ultimately influencing their effectiveness in combating infections [26, 27]. Acute stress triggers a rapid mobilization of neutrophils from the bone marrow and peripheral reservoirs into the bloodstream [11]. In a study with mice, acute stress resulted in an increased infiltration of neutrophils into the lungs following exposure to lipopolysaccharides (LPS), while lymphocytic cell counts were reduced compared to control animals [3]. This response is mediated by catecholamines like adrenaline (epinephrine), which enhance blood circulation and cellular trafficking. As a result, neutrophil effector functions, such as chemotaxis, phagocytosis, and the production of reactive oxygen species (ROS), are enhanced. In contrast, chronic stress can compromise neutrophil function, reduce their phagocytic activity, and induce apoptosis, weakening the overall efficacy of the immune response [11]. In addition, mental stress as exams or bereavement significantly reduced human neutrophils phagocytic activity [28].

Recent studies in murine models have shown that chronic stress promotes cancer metastasis by inducing the release of glucocorticoids, such as cortisol, which stimulate neutrophils to form NETs. These NETs alter the tissue microenvironment, impair the immune response, and facilitate tumor cell adhesion and growth in organs such as the lungs and liver [26, 29].

Although previous studies have examined the influence of stress on NET formation and its impact on tumor progression, the interaction between stress hormones and neutrophil function in pigs, as well as their influence in host–pathogen interactions, has not been thoroughly explored.

Pigs are affected by several endemic diseases across all husbandry systems. In previous studies, we found that degraded NETs promote the growth of NAD-dependent *Pasteurellaceae*, such as *Haemophilus influenzae*, *Actinobacillus pleuropneumoniae (A.pp)*, and *Glaesserella parasuis*, by providing NAD and adenosine [27, 30]. A significant example is the life-threatening lung infection caused by (*A.pp*) [31]*.* Neutrophils are rapidly recruited to the site of infection trying to counteract the *A.pp* infection, although their effector functions are not sufficient to control the infection with *A.pp*, as several in vitro studies show [30, 32]. The virulence factors of *A.pp* and other *Pasteurellaceae* bacteria responsible for the resistance against neutrophils counteract multiple effector functions. The ApX-toxins of *A.pp* are known to kill neutrophils and other leukocytes, which results in prominent NET formation [33–35].Antioxidant systems as oxyR, inhibiting ROS mediated killing and the sap transporter system, preventing bactericidal activity of antimicrobial peptides, are most likely to prevent NET-mediated killing of *A.pp* [36–39]. *A.pp* is known to colonize the tonsils of healthy pigs and can cause peracute death in less than 24 h, although the exact reasons for this rapid progression remain unknown. While the triggering factors for disease outbreaks are widely debated, they are not yet fully understood. Potential triggers may include co-infections with viruses [32, 40] or other lung pathogens [30], as well as stress [41].

Studies have detected stress hormone receptors on neutrophils in various species. Adrenergic receptors were detected in mouse neutrophils isolated from bone marrow[42]. Furthermore, α- and β adrenoreceptors were detected on human neutrophils[43]. The influence of stress hormones on neutrophils was investigated in several studies. One study in fish suggested that neutrophils possess functional glucocorticoid receptors. When activated, these receptors prevent programmed cell death (apoptosis) in neutrophils and enhance their survival during stress [44]. In regard of *A.pp* and stress hormone influence it was shown that the β2-adrenergic receptor modulates activity of porcine alveolar macrophages in response to *A.pp* [45].

Due to the limited research on how stress hormones affect the functionality of immune cells in livestock animals [9], there is a significant gap in understanding the interplay between immune cells, stress hormones, and bacterial pathogens.

Here, we analyzed the influence of stress hormones on neutrophil functions, *A.pp* and the host–pathogen interaction of neutrophils and *A.pp*.

## Material and Methods

### Determination of hormone concentrations

#### Measurement of stress hormones in porcine blood (in vivo)

In total, 72 serum samples from pigs were examined to determine in vivo-relevant concentrations of stress hormones that were tested in this study. Animals of different ages and subjected to various stress factors, including non-infected and infected animals, were to be included in the study. The porcine serum samples were obtained from 44 pigs from two previously published projects with an authority approvement [46, 47]. 1.) 28 piglets (~ 8 weeks old) were infected for 48–96 h with *Streptococcus suis* strains and serum samples were collected pre and post infection (56 samples) [46]. 2.) Serum from 16 healthy pigs (~ 9-month-old) were collected during euthanasia. The euthanasia of these pigs due to other reasons was approved and registered by the local Animal Welfare Officer in accordance with the German Animal Welfare Law under number TiHo-T-2019- 14 [47].

The amount of stress hormones was measured with a pig cortisol ELISA (MBS704168, MyBioSource, Inc. San Diego, USA), a porcine Epinephrine ELISA (MBS260779, MyBioSource) and a Porcine Norepinephrine ELISA (MBS2601585, MyBioSource), following the manufacturer’s instructions. ELISA were analyzed with Multiscan Go (Thermo Scientific N13133) and the analysis software.

### Detection of stress hormone receptors in porcine neutrophils

#### Neutrophil isolation

Blood collection from healthy piglets was registered at the LAVES under No.33.9-42502-05-18A302. In addition, blood was collected in 50 mL lithium-heparin tubes at the slaughterhouse from four healthy pigs during the slaughter process and the sampling complied with relevant regulations. The blood was used latest one hour after blood collection for the neutrophil isolation in the ROS assay with *A.pp* and for the RNA extraction. Purification of porcine neutrophils was conducted from heparinized blood from healthy pigs as previously described [48] using a density gradient with Biocoll® (1.077 g/mL, Bio&SELL GmbH, BSL6115, Nürnberg, Germany). Isolated neutrophils were immediately used in different in vitro assays to determine the neutrophil reaction on stress hormones (with and without infection) or for RNA isolation.

#### RNA expression analysis in porcine neutrophils

RNA was extracted from 5 × 10^5^ neutrophils (two pigs), with the RNeasy MicroKit (Qiagen) as described in the user’s manual including the addition of ß- mercaptoethanol to RLT buffer. RNA quality was tested with a bioanalyzer (RNA 6000 Pico Kit, Agilent) following the manufacturer’s instructions. Only isolates with sufficient integrity (RIN > 7) have been processed further. cDNA has been synthesized from the isolated RNA using GoScript Reverse Transcriptase (A5001, Promega, Madison, Wisconsin, United States) according to the manufacturer’s recommendations. Real-time PCR has been performed using the QuantiTect SYBR Green PCR Kit (204143 Qiagen, Venlo, Netherlands) according to manufacturer’s recommendations. Primers that have been used are listed in Table 1.

**Table 1:** Primer list used in this study

Gene		Sequence	Product size (bp)	Resource
*RPL4*	fwd	CAAGAGTAACTACAACCTTC	122	[49]
	rev	GAACTCTACGATGAATCTTC		
*ACTB*	fwd	AGGCCAACCGTGAGAAGATG	122	This study
	rev	CATGACAATGCCAGTGGTGC		
*NR3C1*	fwd	GTTCCAGAGAACCCCAAGAGTTCA	173	[50]
	rev	TCAAAGGTGCTTTGGTCTGTGGTA		
*NR3C2*	fwd	AAAAGAGCAGTGGAAGGGCA	100	This study
	rev	GAAGTCTGCAGGCAGGACAA		
*ADRB2*	fwd	CGTCATGTCGCTCATTGTCC	204	[45]
	rev	CACCAGAAACTGCCGAAAGTC		
*ADRA1*	fwd	ACCGAACGATGACAAGGAGTG	100	[45]
	rev	GACCAGAATGACCGCCAGAG		

 The PCR product was loaded on a 1% agarose gel to determine the product length and to verify the primer function. 3 µL of Roti Gelstain (3865.1, Carl Roth, Karlsruhe, Germany) was added to 80 mL of gel for DNA visualization. 10 µL PCR product were mixed with 2 µL 6 × TriTrack DNA loading dye (R1161, Thermo Scientific, Waltham Massachusetts, United States). The gel was imaged using the ChemiDoc MP imaging system (BioRad, Hercules, California, United States).

### Determination of neutrophil activity in the presence of stress hormones

#### Stress hormones in in vitro assays

In this study cortisol (Hydrocortisone, H0888, 1g, Sigma-Aldrich, Munich, Germany), epinephrine (( −)-Epinephrine ( +)-bitartrate salt, E4375, 1g, Sigma-Aldrich) and norepinephrine (L-Norepinephrine hydrochloride, 74,480, 100mg, Sigma-Aldrich) were used. They were reconstituted following manufacturer’s instructions (sterile, non-pyrogenic, hypotonic water; 3255.1, Carl Roth®®, Karlsruhe, Germany) and were aliquoted and stored at − 20°C until usage. Each aliquot was used only once. For each stress hormone in the presented in vitro assays, three concentrations were determined based on the measurements from the in vivo samples. The used concentrations are presented in the Supplemental Table 1.

#### Flow cytometry analysis of neutrophils in presence of stress hormones

Intracellular ROS production was measured as described previously [27, 51] with small modifications: Freshly isolated neutrophils (5 × 10^5^ neutrophils/100µL) were incubated for 1 or 3 h (37 °C, 5% CO_2_) in presence of the stress hormones or respective controls. Furthermore, cell size was determined by FSC-A (forward scatter area) and granularity by SSC-A (side scatter area). Data were analyzed with FlowJoTM10.7.1 software (Ashland, OR, USA).

### Determination of neutrophil activity in the presence of stress hormones under *A.pp* infection

#### Fresh growth and cryostocks from *A.pp*

In this study, we used *A.pp* serotype (ST) 2 strain C3656/0271/11 [52]. Fresh self-made pleuropneumonia-like organism (PPLO) medium and agar plates were produced as described previously [27]. *A.pp* ST 2 was grown in PPLO media as described previously [30].

Briefly, *A.pp* was grown until it reached the mid-log phase with an optical density (OD_600nm_) of 0.60 ± 0.01. The bacteria were washed as described previously [30]. Due to slight variations in growth rates, a MOI of 0.5–2 was used.

For preparing *A.pp* cryostocks, fresh grown *A.pp* ST 2 were washed with 1 × PBS LPS free and finally dissolved in a solution of 1 × PBS with 15% glycerol. Aliquots were immediately frozen in liquid nitrogen and stored at -80 °C until usage and used only once.

#### Flow cytometry analysis of neutrophils in presence of stress hormones and *A.pp*

Intracellular ROS production was measured as described previously [27, 51] with small modifications: Freshly isolated neutrophils (5 × 10^5^ neutrophils/100µL) were incubated for 1 (37 °C, 5% CO_2_) in presence of the stress hormones and/or *A.pp* (MOI 0.5–2) or respective controls.

#### NET induction assay with or without *A.pp* and stress hormones

The NET induction assay was conducted as previously described [27] with the following changes: The neutrophils (2 × 10^5^ neutrophils/100µL) were either infected or not with *A.pp* ST2 (MOI = 2) from cryostocks and with and without the three different concentrations from each stress hormone (Supplemental Table 1). As negative control, RPMI 1640 medium was added. As positive control, 100µL methyl-β-cyclodextrin (CD) (10 mM final concentration, C4555; Sigma-Aldrich GmbH) was used. The final volume was 200µL. After adding the stimulus, the samples were centrifuged (370 × *g*, 5 min) and incubated for 3 h at 37 °C, 5% CO_2_. Samples were fixed with paraformaldehyde (4% final concentration) and the plates stored at 4 °C.

#### Visualization and quantification of NETs immunofluorescence images

NETs were stained as previously described [35, 48]. The following primary antibodies were used: A mouse monoclonal antibody (IgG2a) against DNA/histone 1 (MAB3864; Millipore 2,2 mg/mL diluted 1:4000, Billerica, MA, USA) and a polyclonal rabbit anti-human myeloperoxidase antibody (A039829-2 Agilent, Santa Clara, CA, USA, 3.3 mg, 1:309). The following secondary antibodies were used: A goat anti-mouse Alexa 488Plus antibody (1:500, Invitrogen, Carlsbad, CA, USA) and goat anti-rabbit Alexa 633 antibody (1:500, Thermo Scientific, A21070, 2 mg, Waltham, MA, USA). All antibodies were incubated for 1 h at room temperature. For the isotype controls a murine IgG2a (M5409-0.2 mg/mL, 1:364 Sigma Aldrich, Munich, Germany) and rabbit IgG (from rabbit serum, Sigma Aldrich, I5006, 1.16 mg, 1:109) were used instead of the primary antibody.

Samples were recorded using a Leica TCS SP5 AOBS confocal inverted-base fluorescence microscope with a HCX PL APO × 40 0.75–1.25 oil immersion objective.

The NET quantification was conducted as previously described [27]. The cells were counted manually using ImageJ software (version 1.52q, National Institute of Health, Bethesda, MD, USA). A neutrophil was counted as positive if an evident off-shoot of DNA was visible or if at least two of the following criteria were found: enlarged nucleus, decondensed nucleus or blurry rim. The percentage of NET-positive neutrophils was calculated.

#### Transmission electron microscopy (TEM) analysis of NETs

Neutrophils (2 × 10^5^ /100µL) were incubated in a 1.5 mL tube with either RPMI 1640 medium alone or with *A.pp* (MOI = 2) with and without stress hormones (cortisol 60 ng/mL, epinephrine 2400 pg/mL and norepinephrine 4000 pg/mL) for 3 h (37 °C, 5% CO_2_). The concentrations were chosen based on the concentration that showed highest NET induction in the samples analyzed by immunofluorescence microscopy. The final volume per tube was 200µL. After incubation, the samples were centrifuged for 5 min (200 × g) and cell pellets were fixed with 2.5% (vol/vol) glutaraldehyde and stored at 4 °C. For TEM, the fixed and washed samples were stained, analyzed and quantified as previously published [35].

### Determination of *A.pp* survival in the presence of stress hormones and neutrophils

#### Growth of *A.pp* ST 2 in presence of stress hormones

In a 96 well plate, bacteria were grown in presence or absence of three different concentrations of each stress hormone (Supplemental Table 1). A streak out from *A.pp* ST 2 was made. Colony material was suspended into 8 mL of 1 × PBS until the solution was turbid. The solution was adjusted with 1xPBS to OD_600nm_ = 0.5. Subsequently, 10 µL of the bacterial suspension was added in each well. PPLO media was used to complete the final volume of 200 µL in each well. A sterile control of PPLO media with 10 µL 1xPBS instead of bacteria was included in each technical run. Triplicates from each sample were performed. The plate was incubated (37 °C, 5% CO_2_) and the OD_600nm_ was measured in a plate reader (Spark® 30,086,376, TECAN US Inc., Männedorf, Switzerland) over 20 h. After 3 h, the incubation in the plate reader was interrupted and 20 µL were taken from each sample for a serial dilution. This was plated on PPLO agar plates to determine colony forming units (CFU)/ mL after 20 h incubation (37 °C, 5% CO_2_). The 20 h measurement of the 96 well plate was continued.

#### *A.pp* survival assay in presence of stress hormones

Freshly grown *A.pp* ST2 (MOI = 0.5–2) was incubated with 2 × 10^5^/100µL freshly isolated porcine neutrophils in a 48 well plate (Greiner Bio-One, 677102, Kremsmünster, Austria). Stress hormones were added at the following concentrations: cortisol 60 ng/mL, epinephrine 300 pg/mL and norepinephrine 300 pg/mL). The final volume in each well was 200µL. The plates were centrifuged (370 × *g*, 5 min) and were incubated for 3 h (37 °C, 5% CO_2_). Afterwards, 20 µL were taken for a serial dilution in 1 × PBS, followed by plating on PPLO agar plates. Plates were incubated for 20 h (37 °C, 5% CO_2_). Colony-forming units per milliliter (CFU/mL) were calculated, and statistical analyses were performed.

### Statistical analysis

Data were analyzed using Excel 2016 or Excel 365 (Microsoft) and GraphPad Prism version 10.4.1 (627) (GraphPad Software, San Diego, CA, USA). Normal distribution was tested using the D’Agostino & Pearson K2 normality test). Number of conducted independent experiments and differences between groups were tested as indicated in the figure legends (*p < 0.05, **p < 0.01, ***p < 0.001, ****p < 0.0001).

## Results

### Determination of in vivo-relevant stress hormone concentrations for in vitro assays

To analyze the effect of stress hormones on various antimicrobial mechanisms of neutrophils, we first aimed to establish in vivo-relevant concentrations for our in vitro assays. We analyzed 72 serum samples from pigs of different ages to determine the concentrations of cortisol, epinephrine, and norepinephrine. All pigs were housed in the same facility. The younger pigs were only acclimatized for one week, whereas the older pigs were housed for several months until the blood samples were collected. While an experimental *Streptococcus suis* infection did not significantly affected stress hormone levels, we observed significantly higher concentrations of cortisol and epinephrine in younger pigs (approximately 8 weeks old) compared to older pigs (approximately 9 months old) (Supplemental Fig. 1). Based on these findings, we selected three concentrations for the in vitro assays: one close to the mean of the measured values, the maximum value of the measured values, and twice the maximum value of the measured values (refer to Supplemental Table 1).

### Detection of RNA from stress hormone receptors on porcine neutrophils

As we planned in vitro assays with porcine neutrophils and different stress hormones, we started with RNA analysis of porcine neutrophils to ensure that the investigated stress hormones express respective receptors. Cortisol primarily binds to glucocorticoid receptors and in addition can bind mineralocorticoid receptor. By real-time PCR we confirmed the expression of porcine glucocorticoid receptor gene *NR3C1* [53] and the porcine mineralocorticoid receptor gene *NR3C2* [50]. Epinephrine and Norepinephrine can bind to adrenergic receptors. Furthermore, we were able to confirm the expression of porcine alpha (α)1 adrenergic receptor gene *ADRA1* [45] and porcine beta (β) 2 adrenergic receptor gene *ADRB2* [45] (Supplemental Fig. 2). Therefore, we have shown the expression of the RNA of the receptors for the analyzed stress hormones are present in porcine neutrophils.

### ROS production of neutrophils is slightly influenced by stress hormones

**Fig. 1:**
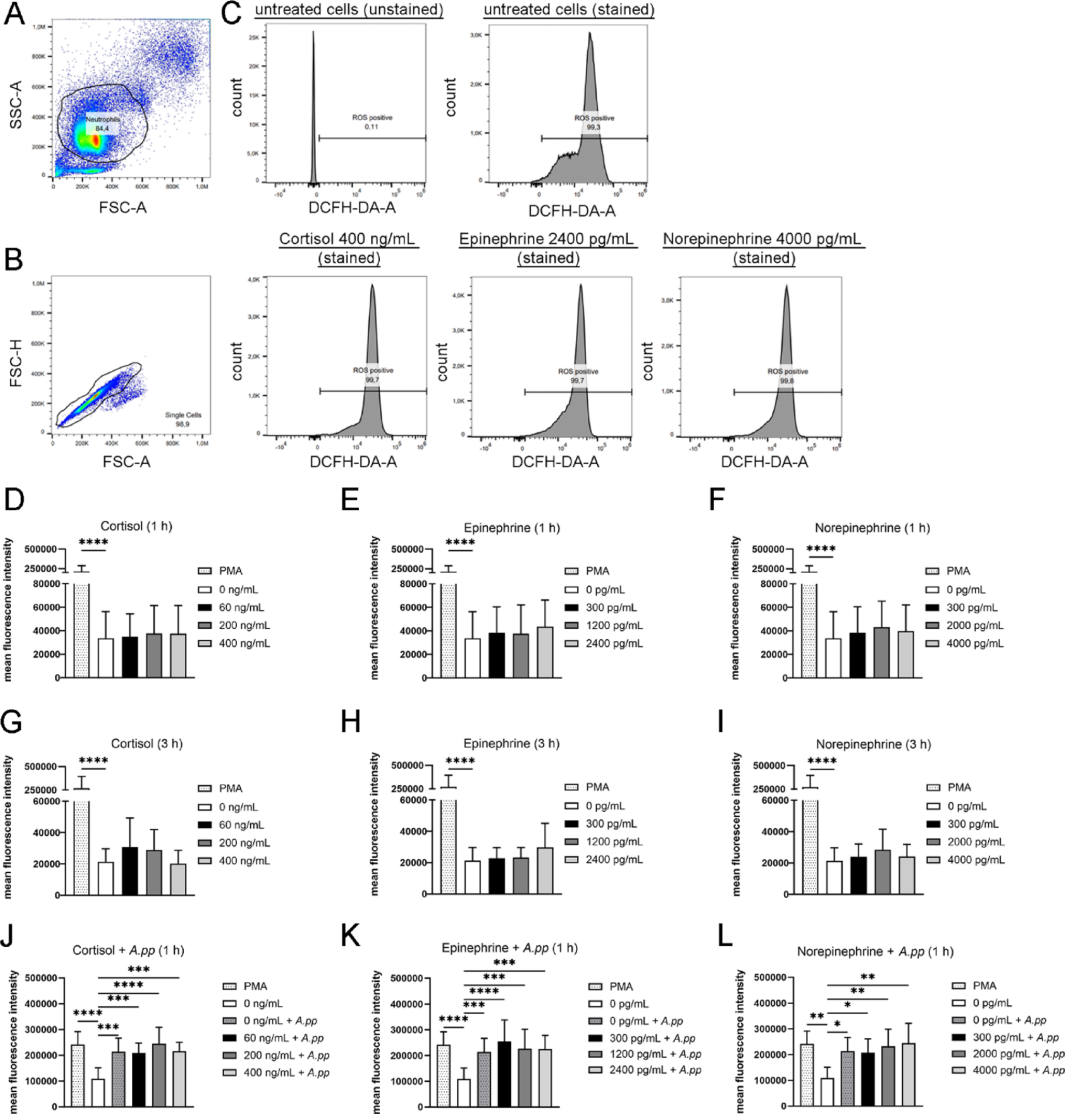
Isolated neutrophils produced increased amounts of ROS upon incubation with stress hormones and *A.pp*. **A-C** The intracellular ROS production was determined by adding 2′7’-dichlorodihydrofluorescin-diacetate (DCFH-DA). The gating strategy for identifying DCF-positive cells via flow cytometry is presented. Neutrophils were gated based on forward scatter area (FSC-A) and side scatter area (SSC-A) characteristics. **B** Single cells from the neutrophil population were gated based on forward scatter area (FSC-A) and forward scatter height (FSC-H). **C** The population of ROS-positive cells was gated according to the unstained control. Example histograms are presented. **D-I** ROS production by neutrophils under stress hormone incubation was assessed. Untreated and stained cells served as controls to determine changes in ROS production during stimulation with stress hormones. **D-F** A dose-dependent enhancement of ROS is detectable after one hour of incubation. **G-I** After three hours of incubation, cortisol concentrations up to 200 ng/mL and epinephrine concentrations up to 2400 pg/mL result in an increase in ROS production. **I** Norepinephrine no longer enhances ROS production. **J-L** Data were analyzed with one-way ANOVA followed by Dunnett’s multiple comparison test and are presented with mean ± SD (**D**–**I** n = 8; **J**–**L** n = 4), (*p ≤ 0.05; **p < 0.01; ***p < 0.001; ****p < 0.0001)

Since ROS represent an early effector response of neutrophils to immunological or physiological stimuli, we examined the influence of stress hormones on ROS release by neutrophils. Assessing ROS production provides a sensitive and rapid means of detecting potential immunomodulatory effects of stress hormones on porcine neutrophils. We determined the ROS production of uninfected and *A.pp*-infected neutrophils in the presence of three different concentrations of cortisol, epinephrine, and norepinephrine (Fig. 1).

After one and three hours in the absence of *A.pp* stress hormones did not significantly induce ROS production. Additionally, no changes in size or granularity were observed in neutrophils after incubation with stress hormones (Supplemental Fig. 3 and 4). An *A.pp* infection significantly induced ROS production, but an additional clear effect due to the presence of stress hormones could not be observed (Fig. 1J–L). However, depending on the stress hormone and the concentration a co-incubation of *A.pp* and stress hormones increased to higher values.

### Increased NET formation with *A.pp* infection and presence of stress hormones

**Fig. 2:**
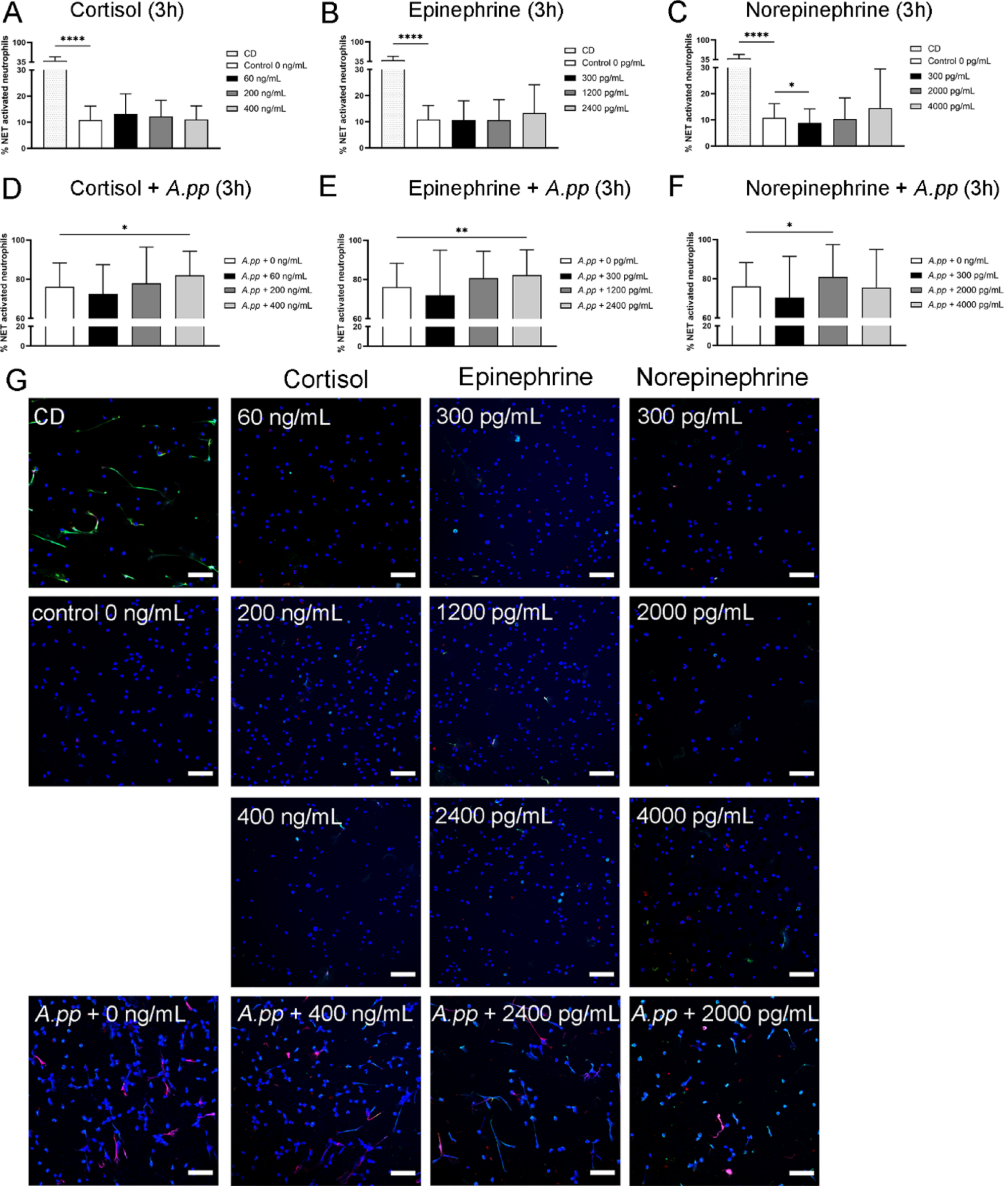
Stress hormones boost the release of NETs during *A.pp* infection in a concentration-dependent manner. **A-F** Neutrophils were stimulated with stress hormones in the absence (**A-C**) and presence (**D-F**) of *A.pp* for three hours. Confocal immunofluorescence images from NET induction assays were analyzed. Data are presented with mean ± SD. Since the data did not pass Shapiro–Wilk normality test, all analyzes were performed using the unpaired Mann–Whitney test (n = 5 independent experiments, with a total of six images per animal; *p < 0.05, **p < 0.01, ****p < 0.0001). **A-C** NET-activated cells were quantified in confocal immunofluorescence images from NET induction assays involving stress hormones without *A.pp*. Methyl-β-cyclodextrin (CD) served as a positive control. No significant NET induction by stress hormones was detected. **D-F** NET-activated cells were quantified in confocal immunofluorescence images from NET induction assays involving stress hormones with *A.pp. ***D** Significant increase in NET formation in presence of 400 ng/mL cortisol (82 ± 12.3%). **E** Significant increase in NET formation in presence of 2400 pg/mL epinephrine (82.3 ± 12.83%). **F** Significant increase in NET formation in presence of 2000 pg/mL cortisol (80.9 ± 19.45%). **G** Example images from confocal immunofluorescence microscopy show neutrophils incubated with various stimuli for three hours. Samples co-incubated with stress hormones and *A.*pp were selected based on the highest NET-induction value. Overlay images are presented (DNA = blue; DNA/Histone1 complexes = green; myeloperoxidase = red; scale bar = 50 µm)

NETs represent another essential antimicrobial mechanism utilized by neutrophils [54]. Since ROS plays a critical role in inducing NET formation, we subsequently evaluated NET release under stress hormone stimulation. Given that we observed increased ROS production in neutrophils stimulated with stress hormones, we suspected an enhancement of NET release. However, using immunofluorescence microscopy, we found only a slight increase in NET-activated neutrophils compared to the negative control across all analyzed concentrations and types of hormones (Fig. 2A–C and G).

*A.pp* induces a high amount of NETs in porcine neutrophils [30, 35]. However, the influence of stress hormones on NET formation during an infection has not been investigated. Using immunofluorescence microscopy, we found that NET formation can be further increased after a three-hour infection with *A.pp* when treated simultaneously with stress hormones, compared to neutrophils infected with *A.pp* alone (Fig. 2). While *A.pp* alone induces NETs in 76 ± 7.88% of neutrophils, the presence of stress hormones amplifies the release depending on the concentration (Cortisol 400 ng/mL: 82 ± 8.8% NET-releasing neutrophils; epinephrine 1200 pg/mL = 80.7 ± 11.4% and 2400 pg/mL = 82.3 ± 5.6% NET-releasing cells; norepinephrine 2000 pg/mL = 80.5 ± 8.3% NET-releasing cells) (Fig. 2D–F and G). Thus, even when NET release was nearly saturated by *A.pp* infection alone, a higher number of NET-releasing neutrophils was observed under the additional influence of stress hormones.

Although stress hormones alone may not highly induce the formation of NETs, they may contribute to an exacerbated reaction as response to a secondary stimulus, like a pathogen.

### Stress hormones do not influence *A.pp* growth

To further investigate whether the observed increase in NET release is a direct result of stress hormone stimulation or an indirect consequence of enhanced *A.pp* growth under stress conditions, we conducted experiments to assess the effect of stress hormones on *A.pp* proliferation in the absence of neutrophils. However, neither growth curves measuring optical density nor the determination of colony-forming units showed any influence of stress hormones on the growth of *A.pp* (Supplemental Fig. 5).

### Stress hormones do not enhance the growth of *A.pp* in the presence of neutrophils

**Fig. 3:**
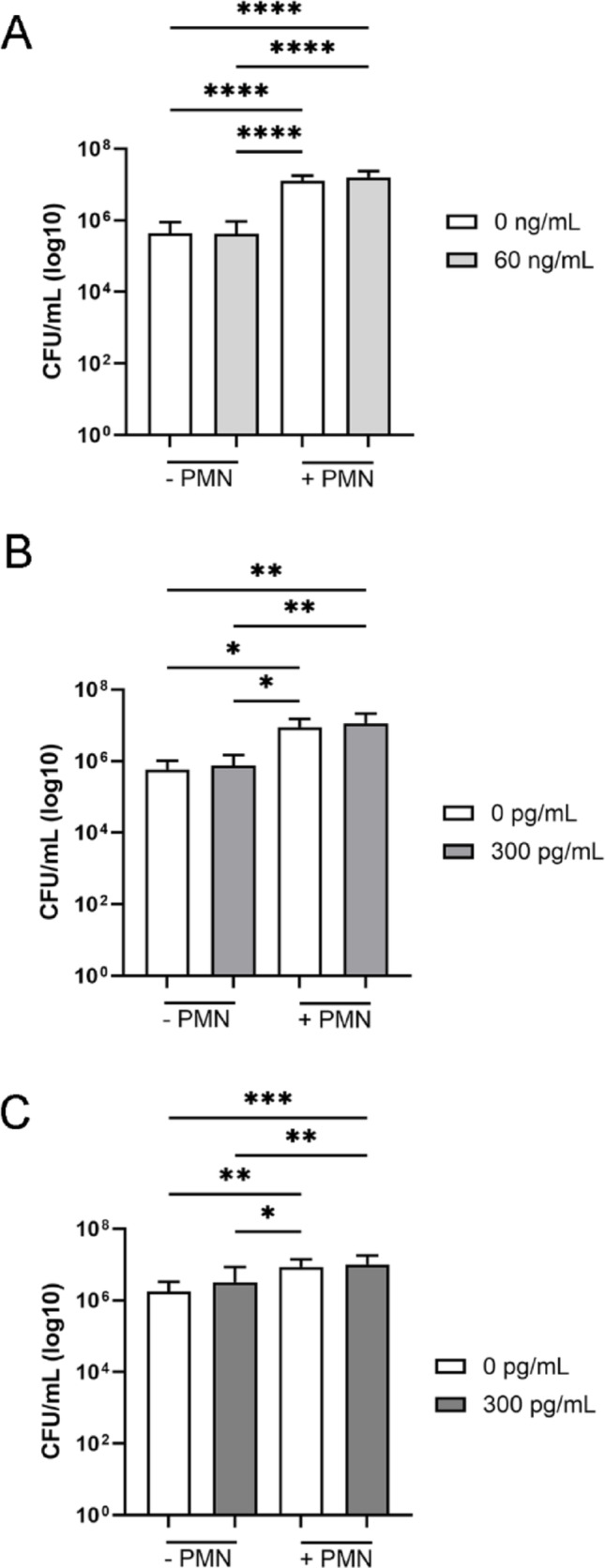
Stress hormones do not alter the neutrophil-mediated growth advantage of *A.pp* in presence of neutrophils. **A-C** Effect of stress hormones on *A.pp* survival in the presence or absence of porcine neutrophils (PMNs): Stress hormones did not affect bacterial growth. However, in the presence of neutrophils, bacterial survival increased significantly compared to the condition without neutrophils, confirming that *A.pp* benefits from the presence of neutrophils for its growth. **A** Samples were treated with cortisol. **B** Samples were treated with epinephrine. **C** Samples were treated with norepinephrine. Data were analyzed with one-way ANOVA followed by Tukey’s multiple comparison test and are presented with mean ± SD (n = 8), (*p ≤ 0.05; **p < 0.01; ***p < 0.001; ****p < 0.0001)

*A.pp* not only induces significant NET release in neutrophils, but it is also known to exploit these degraded NETs for its growth by utilizing NAD and adenosine [30]. Observing that neutrophils respond to stress by releasing more NETs in the presence of *A.pp*, we investigated the growth of *A.pp* when stress-hormone-treated neutrophils are present. We confirmed that neutrophils without stress-hormones treatment enhance the growth of *A.pp* [30] (Fig. 3 and Supplemental Fig. 6). This phenotype is unchanged with stress hormone treated neutrophils. These data indicate that although the reaction of neutrophils is influenced by stress hormones, the growth of *A.pp* is not significantly changed. *A.pp* is able to benefit from activated neutrophils independently of the presence of stress hormones, as *A.pp* strongly induces NETs [30].

### Increased vital NET formation following incubation with stress hormones

**Fig. 4:**
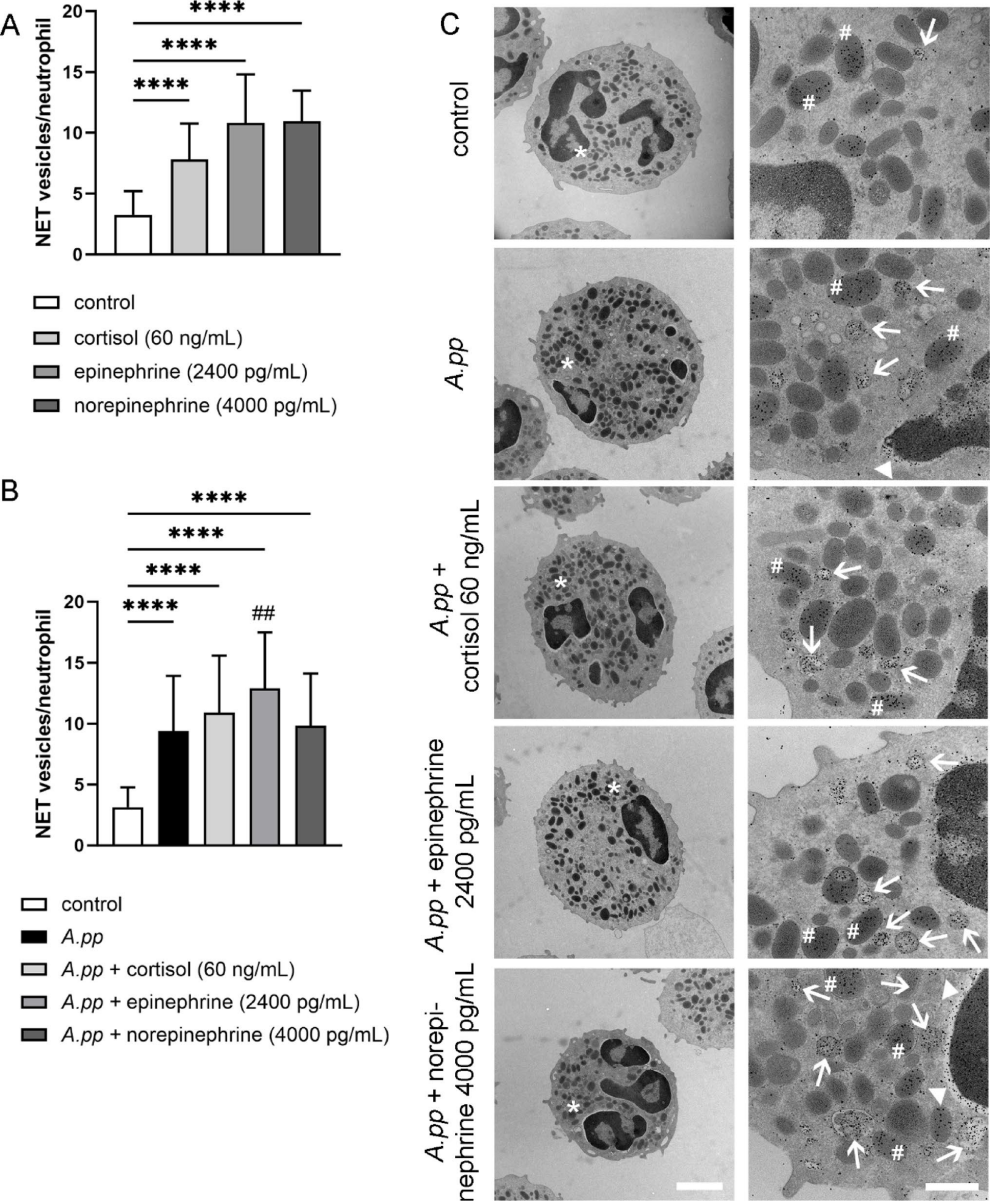
Stress hormones induce vesicular NETs in neutrophils. **A-B** Statistical analysis of the TEM images: A total of 30 cellular profiles from randomly selected fields on the thin sections were analyzed per sample. **A** A significantly higher number of NET vesicles, indicative of "vital NETosis," were identified in neutrophils, with the highest numbers observed in those treated with epinephrine (2400 pg/mL) and norepinephrine (4000 pg/mL). Data were analyzed with one-way ANOVA, followed by Dunnett’s multiple comparison test (*p* = 0.0001; n = 30 cellular profiles) and presented with mean ± SD (p**** < 0.0001). **B** Neutrophils infected with *A.pp* and stimulated with stress hormones showed a significantly higher number of NET vesicles indicative of "vital NETosis," particularly under stimulation with epinephrine at 2400 pg/mL. Data were analyzed with ordinary one-way ANOVA, followed by Dunnett’s multiple comparison test (*p* = 0.0001; n = 30 cellular profiles) and presented with mean ± SD (p**** < 0.0001). In addition, an unpaired one-tailed Student’s t-test was calculated between the *A.pp*-treated sample and each sample treated with both *A.pp* and a stresshormone (p^##^ < 0.001). **C** Representative TEM images of porcine neutrophils after three hours of incubation with stress hormones and *A.pp*, with RPMI included as a negative control. In the overview images (left panel), the white star indicates the areas that have been zoomed in. The zoomed-in images (right panel) show immune gold labeling used to identify NET vesicles (indicated by white arrows; 5 nm gold labeling = H3-cit and 10 nm gold labeling = NE). White hash marks indicate neutrophil elastase (NE)-positive granules. Membrane blebbing of the nucleus, marked by NE/H3-cit positive gold-labeled (white arrowheads), was observed in some cases. The scale bars are: 2 µm (left) and 500 nm (right)

Given that a significant increase in NET release had previously been observed under stress hormone stimulation, we conducted further analysis of neutrophils using transmission electron microscopy (TEM) to better characterize the nature of NET formation with a focus on vesicular NETs under these conditions. We selected the concentration of each hormone that resulted in the highest number of NET-activated cells according to the immune fluorescence microscopy analysis (cortisol 60 ng/mL = 13.15 ± 6.1%; epinephrine 2400 pg/mL = 13.35 ± 6.2%; norepinephrine 4000 pg/mL = 14.55 ± 11%). Indeed, by TEM we could observe that these concentrations induce “vital” NETosis, indicated by formation of vesicles with NET components as well as membrane blebbing of the nuclei [18] (Fig. 4).

Neutrophils incubated with cortisol (60 ng/mL), epinephrine (2400 pg/mL), or norepinephrine (4000 pg/mL) exhibited a higher number of NET vesicles (indicated by arrows) compared to the control condition, consistent with “vital” NETosis. Quantification of NET vesicles per neutrophil revealed a significant increase under all stress hormone treatments (Fig. 4A). During *A.pp* infection (Fig. 4B), neutrophils demonstrated an elevated number of NET vesicles relative to uninfected controls, confirming that this bacterial infection promotes NET release, including vesicular NETs. Among the stress hormones, only epinephrine stimulation showed a significant increase in the number of NET vesicles during *A.pp* infection, whereas cortisol and norepinephrine did not lead to significant changes. These results demonstrate that stress hormones can enhance vesicular NET formation in neutrophils and suggest a potential hormone-specific modulation of vital NETosis. This modulation, however, shows a reduced effect in combination with another strong NET-inducing stimulus like *A.pp*.

## Discussion

Stress has been identified as a critical factor modulating immune responses in both animals and humans [4, 55]. It is discussed whether the frequent occurrence of *A.pp* outbreaks are maybe influenced by stress [56]. In this context, stress hormones such as cortisol, epinephrine, and norepinephrine could interfere with the innate immune system, particularly neutrophils, which play a key role in the initial response to bacterial infections. These hormones may affect 1) the functionality of neutrophils, 2) the ability to control bacterial growth, or 3) both simultaneously. This implies that stress-induced modulation of innate immunity may compromise host defense mechanisms. The aim of our study was therefore to investigate how physiologically relevant concentrations of stress hormones affect key antimicrobial functions of porcine neutrophils in vitro and/or *A.pp* growth in the presence or absence of neutrophils in the timeframe of an acute stress scenario.

In summary our data show that the growth of *A.pp* was not influenced by the presence of stress hormones (Supplemental Fig. 5), which aligns with a previously published study on different field isolates of *A.pp*. In that study, epinephrine did not affect the growth of nine different isolates from three different serotypes [57]. However, one study described that when *A.pp* is grown in a chemically defined medium, in contrast to rich medium, its growth is promoted by stress hormones due to their influence on iron metabolism [58]. In our study, we conducted the growth experiments using an enriched medium (PPLO). Nevertheless, at least in the presence of RPMI, a medium in which *A.pp* does not grow, no growth-boost was observed from the tested stress hormones (Fig. 3). We cannot exclude the possibility of growth promotion in the presence of a chemically defined medium or under in vivo conditions. Future research could clarify whether, for example, in the presence of natural environments, such as bronchoalveolar lavage fluid (BALF), stress hormones could boost the growth of *A.pp*. This could be particularly interesting, as our group recently observed a growth boost in BALF for *A.pp*, depending on the health status of the pig (e.g., if infected with Influenza A virus) [32].

For other bacteria, such as *Streptococcus pneumoniae* [59] and *Listeria monocytogenes* [60], a growth-promoting effect by stress hormones has been described. Nonetheless, other studies report growth inhibition by stress hormones, for instance, in *Staphylococcus aureus* [61]. Therefore, the results presented in this study cannot be directly transferred to other host–pathogen interactions and investigations are needed pathogen and environment dependent.

As second factor that might be influenced by stress hormones, we analyzed the influence on neutrophil functions. Indeed, we identified the expression of receptors for cortisol, epinephrine and norepinephrine on RNA levels in porcine neutrophils (Supplemental Fig. 2) and further that stress hormones slightly influence the production of ROS in a concentration dependent manner, especially in presence of *A.pp* (Fig. 1). In good correlation to our data, methylprednisolone, a glucocorticoid as cortisol, enhances the ROS production in canine neutrophils concentration dependent [51]. On the other hand, a clinical study in humans with an intravenous application of hydrocortisone, the medical treatment form of cortisol, showed an inhibition of ROS production after one and two hours after treatment using an ex vivo detection method with a luminometer and isolated neutrophils [62]. The inhibition of ROS production gradually disappeared, and the ROS levels returned to those measured before the application. This result might depend on the high concentration of hydrocortisone (100 mg/per treatment). If we assume an average blood volume of five liters for the participants in the study, this dose will correspond to 20,000 ng/mL of cortisol. This would be 50 times higher than the highest concentration tested in this study. Hydrocortisone is a fast-acting glucocorticoid that starts after one to two minutes to act after an intravenous application with a half-life of around 1.7 h [63]. In addition, it is described that higher doses can lead to increased clearance and increased volume of distribution, likely due to protein-binding saturation [64]. Therefore, in vivo, various influencing factors can affect the action of cortisol, which can make a direct comparison between in vivo and in vitro data difficult.

While we cannot exclude the occurrence of higher stress hormone concentrations during acute or extreme stress responses, our study focused on physiologically relevant levels based on in vivo measurements obtained from pigs under respective husbandry conditions. Compared to the values available in the literature, the values we measured in porcine blood are comparable. High concentrations were described under acute stress (e.g. transport) [65, 66]. In addition, the highest concentrations used (concentration 3) in our study were also observed in pigs subjected to a snare restraint, with even higher concentrations being measured [67]. Therefore, all used concentrations reflect possible in vivo concentrations. The blood we have used in our study to isolate neutrophils derived on the one hand from healthy animals housed in the university intern swine clinic and on the other hand from animals in a local slaughterhouse. It is therefore conceivable that the animals and thereby also the neutrophils in the blood were exposed to stress hormones in the time before blood sampling. Additionally, it is conceivable that the levels of stress vary between the animals in the clinic and the slaughterhouse. To account for that, all experiments were done in parallel with matched samples, so that in each individual experiment the respective treated samples and controls derive from the same animal. We did not use blood from clinic animals and slaughterhouse animals within one experiment. All effects that may be caused by pre-exposure to stress hormones are therefore excluded by the study design. The experiment investigating ROS under the presence of *A.pp* (Fig. 1J–L) was conducted using blood from animals from the slaughterhouse, while the experiment without *A.pp* (Fig. 1D–I) were performed with animals from the clinic. It is observable that the baseline of ROS production in the slaughterhouse animals is vastly increased, however the ROS production in the positive control reaches the same value in both experiments. One hypothetical assumption is that the animals were exposed to stress before slaughter [68]. However, a distinct phenotype caused by the exposure to PMA, or *A.pp* and stress hormones is nevertheless detectable.

With respect to epinephrine, we observed a dose-dependent enhancement of ROS release. A study using neutrophils from rats found that applying 5 nM adrenaline (~ 900 pg/mL epinephrine, which corresponds to the middle concentration used in our study) in the presence of glucose reduced superoxide production [69]. However, this effect was only marginal when glutamine was present instead of glucose. This also suggests that it is important in the future to conduct studies on the influence of stress hormones on neutrophils in a natural milieu. Nonetheless, new, partially unknown factors such as cytokines could also have an impact and could complicate the analysis.

Another point we observed is that after three hours, norepinephrine no longer induces ROS, whereas epinephrine, at least at the highest concentration, does (Fig. 1). Both norepinephrine and epinephrine are highly susceptible to oxidation. In the presence of oxygen, they undergo rapid autoxidation, forming quinones and other degradation products, which leads to a loss of biological activity [70]. Epinephrine is slightly more stable against oxidation compared to norepinephrine. This could also explain the observed phenotypes. Future studies could include antioxidants, as they are added in pharmaceutical preparations to prevent oxidation [71].

Reactive oxygen species (ROS) plays a key role in the antimicrobial functions of neutrophils [72] and have been linked to the induction of NET formation [73, 74]. In our study, *A.pp* infection alone led to a clear induction of NETs in porcine neutrophils (Fig. 2). Co-stimulation with stress hormones further enhanced NET formation to a limited extent, suggesting a partial additive effect. Interestingly, treatment with stress hormones alone did not induce conventional extracellular NET structures, despite significantly increasing ROS production (Fig. 1).

At the same time, we observed that stress hormones alone triggered the release of vesicular (vital) NETs (Fig. 4), a form of NET release that preserves neutrophil viability. This finding points to a possible link between stress hormone–induced ROS production and the formation of vesicular NETs. However, the mechanistic relationship between ROS and vesicular NET release remains unclear.

To our knowledge, this is the first study to demonstrate that all three stress hormones analyzed—cortisol, epinephrine, and norepinephrine—can induce vital NET formation in porcine neutrophils. Notably, the catecholamines appeared more potent than cortisol in this context, indicating that even short-term stress exposure may modulate neutrophil function via the induction of vesicular NETs. Future studies are needed to further characterize the regulation, composition, and functional relevance of vesicular NETs, which remain poorly understood to date. Although vital NETs are considered to be as antimicrobially active as suicidal NETs and allow the neutrophil to remain intact after formation, neutrophils that have undergone vital NET formation are unable to form NETs again upon subsequent pathogen contact [19]. Therefore, a latent stimulation with stress hormones will still impair a future immune reaction against pathogens. The data from Figs. 2 and 4 show an increase in NET-formation, vital and suicidal, during infectious scenarios in combination with stress hormones. These increases may appear minor, but the actual biological relevance of acute stress on NET-formation may be only determined in an in vivo study or an in vitro infection scenario with a NET-susceptible pathogen.

Despite the observed increase in ROS production and vesicular NET formation, *A.pp* remains highly resistant to neutrophil-mediated killing. Under the given assay conditions, the host–pathogen interaction between porcine neutrophils and *A.pp* appears largely unaffected. The inability of vesicular NETs to eliminate *A.pp* suggests that this form of NET release may not contribute to bacterial clearance and could even have detrimental consequences for the host, such as prolonged inflammation or tissue damage. Since other bacterial pathogens are more sensitive to NET-mediated killing, further studies are warranted to assess whether stress hormone–induced changes in neutrophil function might alter host–pathogen interactions in a pathogen-specific manner. Other pathogens may be more effectively eliminated by neutrophils under stress conditions. For example, a study in mice showed that elevated corticosteroid levels following morphine administration enhanced antibacterial immunity against *Listeria monocytogenes* and *Streptococcus pneumoniae* [75], highlighting that the immune response of neutrophils can vary depending on the pathogen and the hormonal environment.

In contrast, *A.pp* is a well-known neutrophil survivor that induces strong NET formation but remains resistant to NET-mediated killing [30]. Consistent with this, our data show that *A.pp* not only survives in the presence of neutrophils and ROS, but may even benefit from their presence, as indicated by increased bacterial growth under co-culture conditions (Fig. 3). Notably, none of the tested stress hormones significantly altered this interaction. Thus, *A.pp* appears largely unaffected by stress hormone–induced changes in neutrophil activity, including the formation of vesicular (vital) NETs.

These findings suggest that, under the conditions tested, neither the host nor *A.pp* experiences a clear advantage or disadvantage due to short term stress hormone exposure. However, for other bacterial species that are more susceptible to ROS or NET-mediated killing, stress hormones could substantially alter the course of infection. In addition, a potential rebound effect after the resolution of stress cannot be excluded, as temporarily enhanced neutrophil activity may lead to functional exhaustion. When discussing the effect of stress on immune functions, it should always be clarified if short term or long term stress is investigated, as short term stress may enhance immune functions, while long term stress is generally seen as detrimental. Future studies should explore these dynamics in a pathogen-specific context to better understand how stress influences early immune responses.

The study has strengths and weaknesses. On the one hand, it provides valuable analysis for better understanding the influence of in vivo relevant stress hormone concentrations on neutrophil granulocytes, which were systematically examined in this study. However, most observed phenotypes are not strongly pronounced. For example, in the quantification of NETs, there is only a slight increase compared to the negative control. Nonetheless, it cannot be ruled out that in a more complex system, such as the whole-body regarding interaction of multiple cells, the effect might be more pronounced. Moreover, this study did not investigate whether modulators released during stress might impact or modulate neutrophil activity. Examples of such modulators include neuropeptides like neurokinin A and substance P. Future studies could include these substances.

In this study, all assays were conducted with stress hormone concentrations that are relevant in the blood and were determined in this study. Based on the assumption that neutrophils circulate in the bloodstream, come into contact with stress hormones there, and then infiltrate tissues. Therefore, any conclusions drawn from this study are primarily limited to this assumption. The potential influence of stress hormones when a neutrophil migrates into tissue to combat an infection and only then comes into contact with stress hormones was not part of this study. To address this question, in-depth studies would be necessary to test organ-specific and stress hormone-dependent concentrations of stress hormones. Data on the bioavailability of stress hormones in tissues are very limited. However, based on a study in mice, it is indeed possible that the concentration of stress hormones in tissues is higher than that in the blood [76]. Future studies could investigate these aspects in more detail.

Furthermore, in this study, we did not investigate the effect of opsonizing antibodies on the killing of *A.pp* by neutrophils in the presence of stress hormones. However, since pigs with protective antibodies against the respective serotypes are rarely affected by severe cases [77], it is suggested that the influence of stress hormones is minimal. Future studies could verify whether the survival of *A.pp* in the presence of stress hormones and neutrophils differs when sera from vaccinated and non-vaccinated animals are added. Comparable assays showed significant killing of *A.pp* when convalescent sera are present [78].

In conclusion, the finding that stress hormones induce viable (vesicular) NETs in porcine neutrophils is highly relevant, as this form of NET release on the one hand preserves cellular functions such as phagocytosis, but on the other hand depletes NET-forming capacities for coming infections. This observation provides new insights into the role of neutrophils in the innate immune response under stress conditions and contributes to a better understanding of host–pathogen interactions in stressed individuals. Although *A.pp* itself does not appear to be significantly affected by stress hormone–induced changes in neutrophil function, our study provides valuable insights into how stress modulates key innate immune mechanisms. By establishing physiologically relevant hormone concentrations and systematically analyzing their effects on neutrophil responses, we lay the groundwork for future studies exploring pathogen-specific immune modulation under stress. This is particularly relevant in livestock production, where stress is common and can influence disease dynamics in complex ways.

## Supplementary Information

Below is the link to the electronic supplementary material.

Supplementary Material 1

## Data Availability

The authors confirm that the data supporting the findings of this study are available within the published article. Raw data were generated at the Department of Biochemistry, University of Veterinary Medicine Hannover, Foundation, Hannover, Germany and at the Microscopy Facility at the Department of Biology, Lund University. Derived data supporting the findings of this study are available from the corresponding author NdB.
