# Supplementary material for: Immunomodulatory effect of stress hormones on porcine neutrophil functions during Actinobacillus pleuropneumoniae infection
Source: Med Microbiol Immunol. 2026 Jul 27;215(1):21. doi: 10.1007/s00430-026-00881-3 (PMC13407460; doi:10.1007/s00430-026-00881-3)
Supplement: Supplementary file 1 — Supplementary Material 1 [file 430_2026_881_MOESM1_ESM.pdf]

Supplemental data

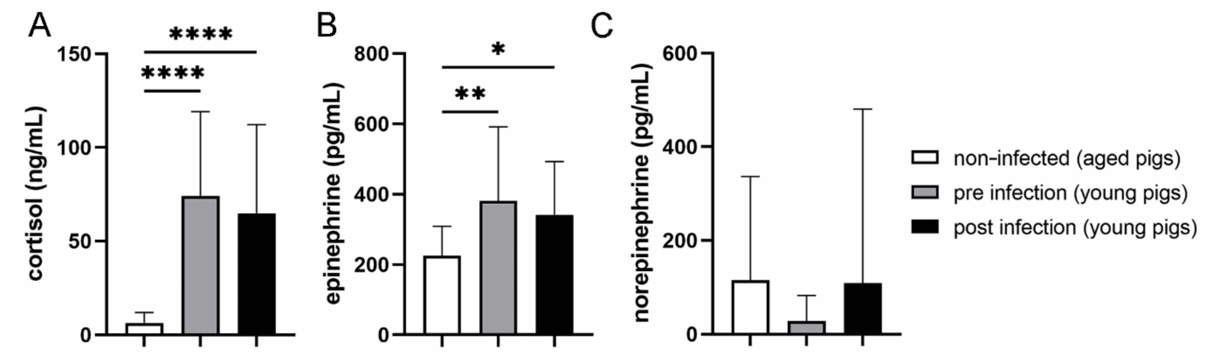

Supplemental Figure 1. Determination of *in vivo* relevant stress hormone levels in aged pigs (~9 month) and young piglets (8 weeks).

A-C The stress hormone levels were measured in serum samples of non-infected aged pigs (n=16) and piglets before and 48-96 hours after intranasal infection with *S. suis* (each group n=28). **A-B** Significantly higher cortisol (*p*<0.0001) and epinephrine (*p*=0.0042) levels were measured in the samples from young piglets, but no differences were identified before and after an infection. **C** Norepinephrine was detected only in 13 out of 72 samples with a high inter-individual variation. No significant difference between groups were observed (*p*=0.0842). All data are presented with mean ±SD and were analyzed with the Shapiro-Wilk test for normal distribution and afterwards with a one-way ANOVA followed by Kruskal-Wallis test (**p*≤0.05, ***p*<0.01, *****p*<0.0001).

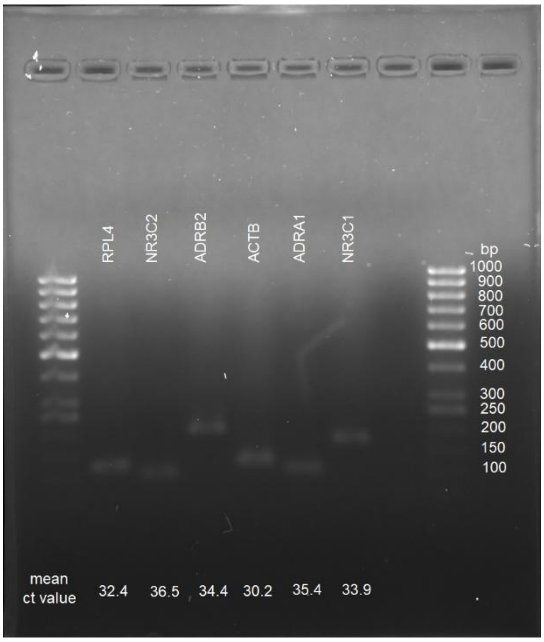

Supplemental Figure 2 Detection of stress hormone receptors on porcine neutrophils by real time PCR.

The real-time PCR product was loaded on a 1 % TBE gel to determine the product length and to verify the primer function. Housekeeping genes: *RPL4* = Ribosomal protein L4 (accession no. DQ845176); *ACTB* **=** Beta-actin (accession no. DQ845171). Stress hormone receptors: *NR3C1* = Glucocorticoid receptor (accession no. AY779185); *NR3C2* = porcine mineralocorticoid receptor (accession no. M36074); *ADRA1* = alpha (α)1 adrenergic receptor (accession no. AJ251727) and *ADRB2* = porcine beta (β) 2 adrenergic receptor (accession no. AF000134). RNA was isolated from two pigs. The ct value (mean out of two technical runs) is presented.

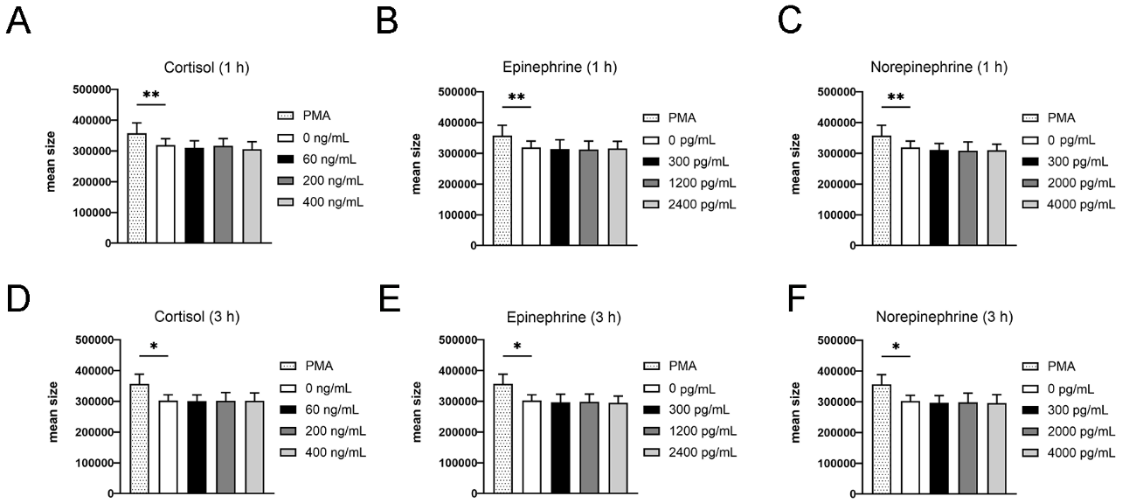

Supplemental Figure 3 Morphology (size) of neutrophils after incubation with stress hormones

Harvested neutrophils were analyzed by flow cytometry after incubation with stress hormones for one and three hours. **A-F** Mean size of neutrophils, based on forward scatter/area of intensity (FSC-A), was significantly different between PMA (phorbol-12-myristate-13-acetate) stimulated and unstimulated neutrophils (n = 8, one-way ANOVA followed by Dunnett's multiple comparison test). No significant differences were observed between unstimulated and stress hormone-stimulated neutrophils. P values *p < 0.05 and **p < 0.01 were considered significant.

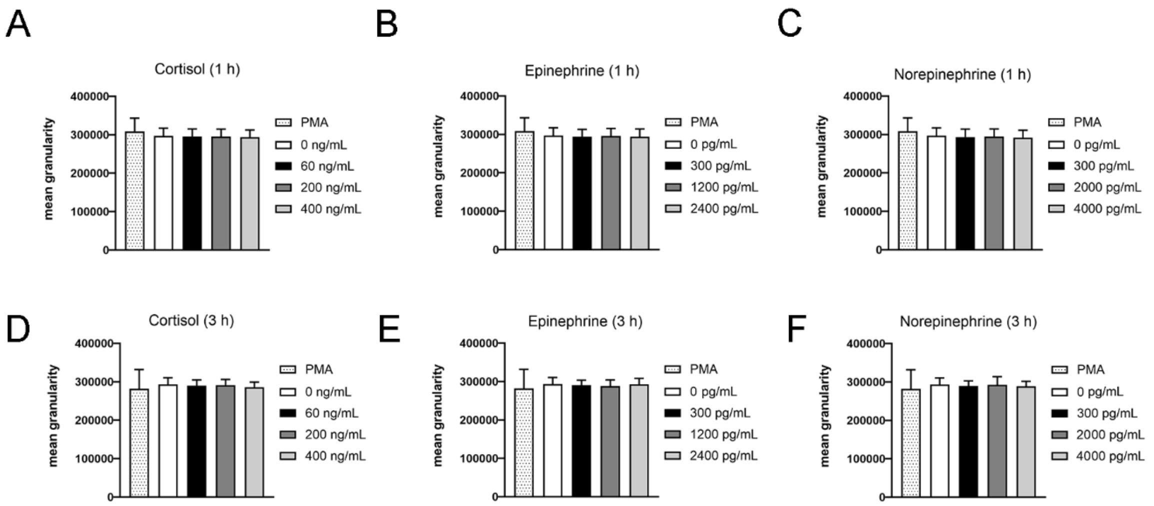

Supplemental Figure 4 Morphology (granularity) of neutrophils after incubation with stress hormones

Harvested neutrophils were analyzed by flow cytometry after incubation for one and three hours with stress hormones. **A-F** Mean granularity of neutrophils based on sideward scatter/area of intensity (SSC-A) was not significantly different (n = 8, one-way ANOVA).

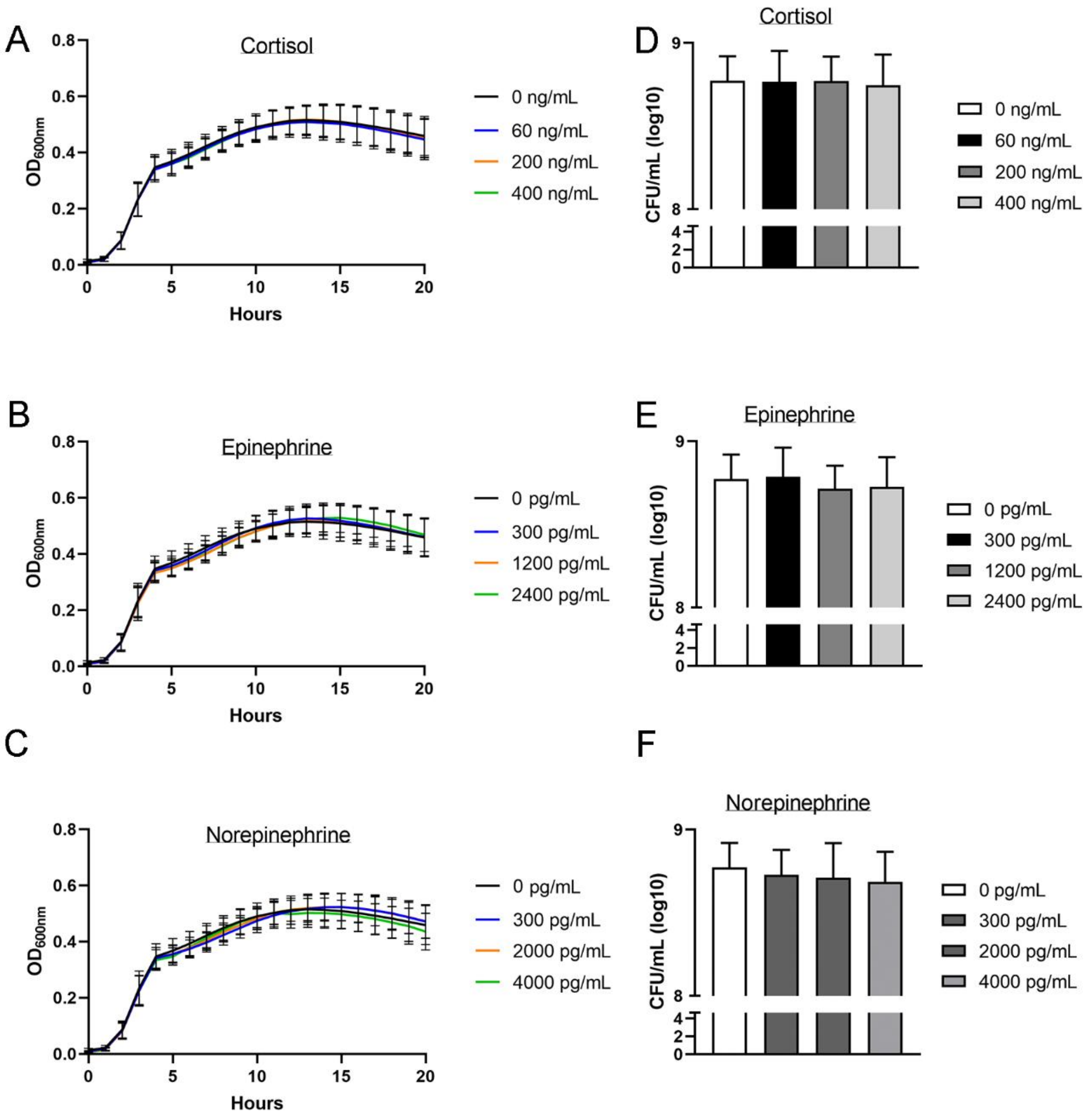

Supplemental Figure 5 The growth of *A.pp* is not influenced by the presence of stress hormones. A-C The growth of *A.pp* was assessed by measuring optical density (OD) over 20 hours, with all data are presented as mean ± SD. Across different stress hormone concentrations, growth was not statistically different. Data were analyzed using a two-way ANOVA (n=7 independent technical runs). **D-F** After three hours of incubation colony forming units (CFU/mL) were determined by plating and results are presented on a logarithmic scale. The CFU/mL were not significantly affected by the three concentrations of the stress hormones analyzed. All data were tested for normal distribution and then analyzed with a one-way ANOVA, followed by Dunnett’s test comparing them to the sample without stress hormones (n=6). All data are presented with mean ± SD.

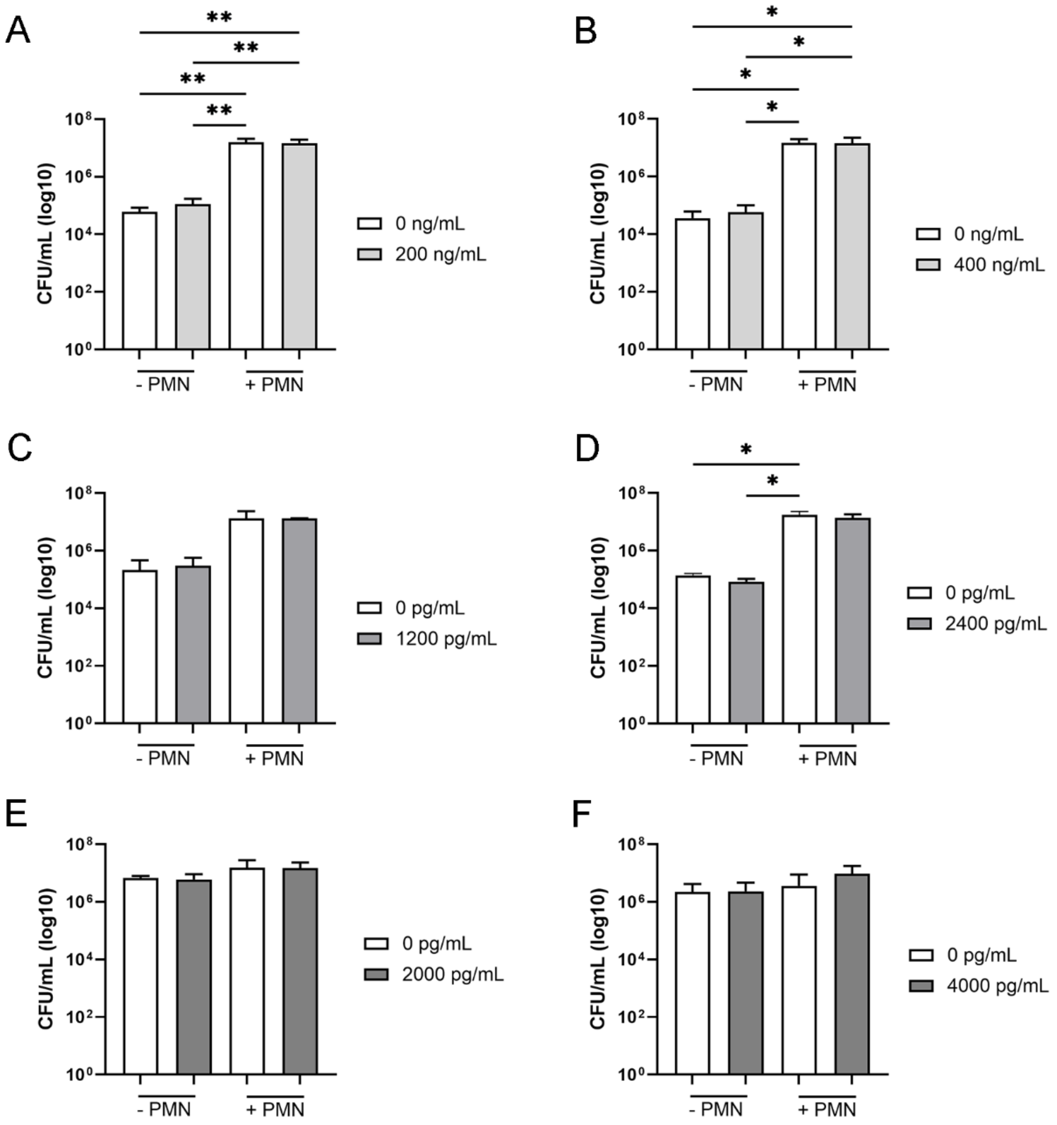

Supplemental Figure 6 Stress hormones do not alter the neutrophil-mediated growth advantage of *A.pp* in presence of neutrophils. A-F Effect of stress hormones on *A.pp* survival in the presence or absence of porcine neutrophils (PMNs): Stress hormones did not affect bacterial growth. However, in the presence of neutrophils, bacterial survival increased compared to the condition without neutrophils, confirming that *A.pp* benefits from the presence of neutrophils for its growth. **A-B** Samples were treated with cortisol (n=3). **C-D** Samples were treated with epinephrine (n=2). **E-F** Samples were treated with norepinephrine (n=3). Data were analyzed with one-way ANOVA followed by Tukey's multiple comparison test and are presented with mean ±SD, (*$p \leq 0.05$; **$p < 0.01$).

Supplemental Table 1. Concentrations of stress hormones selected for the *in vitro* experiments. The mean and maximum values were calculated and based on these values three concentrations for *in vitro* assays were chosen (concentration close to mean, maximum value, and twice maximum value).

		**Cortisol (ng/mL)**	**Epinephrine (pg/mL)**	**Norepinephrine (pg/mL)**
**Concentrations measured in porcine blood**	Minimum	0.49	94.43	12*
	Maximum	186.3	1234	1933
	Mean	53.45	331.2	381.5 **
**Concentrations selected for the *in vitro* experiments**	Concentration 1	60	300	300
	Concentration 2	200	1200	2000
	Concentration 3	400	2400	4000

* 59 from 72 samples were lower the detection limit (12 pg/mL) of the ELISA

** mean of all 72 samples = 78.72 pg/mL (samples lower detection limit set to 12pg/mL), the presented mean is calculated with the 13 samples above the detection limit
